# Construction of the Competency Index System for Nursing Staff in Tertiary General Hospitals to Respond to Emerging Infectious Diseases

**DOI:** 10.3390/healthcare13050476

**Published:** 2025-02-22

**Authors:** Minye Li, Chen Zhi, Dianjie Chen, Zhengwen Ma, Min Zhu, Yanlan Ma, Hui Ma

**Affiliations:** 1 Department of Nursing, Chinese PLA General Hospital, Beijing 100853, China; myli233@163.com (M.L.); longkang1@163.com (C.Z.); wintertonghua@163.com (D.C.); 2 School of Nursing, Southern Medical University, Guangzhou 510515, China; 16699196513@163.com; 3 Department of Disease Control and Prevention, 6th Medical Center of PLA General Hospital, Beijing 100048, China; 18911003710@163.com; 4 Medical Service Training Center, Chinese PLA General Hospital, Beijing 100853, China; mayl301@126.com

**Keywords:** tertiary general hospitals, nursing staff, emerging infectious diseases, competency, index system

## Abstract

**Background:** The outbreak of emerging infectious diseases represents a crisis event that poses a threat to human beings. Nursing staff in the tertiary hospital, especially clinical nurses and nursing managers, serve as the frontline personnel in combating this crisis. However, there is a lack of clarity regarding the specific competencies required of them to respond to these emerging infectious diseases. **Methods:** The literature review, semi-structured interviews, and group discussion were adopted to collect and analyze the competency index. The Delphi technique was used for the examination and construction of the index system. **Results:** The development of the competency index system was a collaborative effort that involved 18 experts from eight provinces and cities across China. The system includes competency indexes for two types of nursing staff: clinical nurses and clinical nursing managers. Specifically, the competency index system for clinical nurses and nursing managers in tertiary general hospitals to respond to emerging infectious diseases comprises three first-level indexes. For clinical nurses, there are 20 second-level indexes and 65 third-level indexes, while for clinical nursing managers, there are 21 second-level indexes and 68 third-level indexes. **Conclusions:** The constructed competency index system for nursing staff in tertiary general hospitals to respond to emerging infectious diseases is scientific, reliable, comprehensive, and specific and may provide reference for nursing leaders to develop competency training programs for nursing emerging infectious diseases.

## 1. Introduction

Emerging infectious diseases (EIDs) are characterized by a high risk of infection, rapid transmission, a wide range of infections, and a high transmission capacity, resulting in a significant impact on global public health [1]. The pathogens in EIDs account for at least 15% of all human pathogens [2]. The 1918 flu pandemic killed more than 50 million people a century ago. In recent decades, EIDs have also posed serious threats. These include SARS (2003), H1N1 (2009), MERS (2012), Zika (2015), and COVID-19 (2019). They have raged in countries and even around the world, seriously affecting human health and social stability [3]. A considerable number of newly emerging infectious diseases have been traced back to viral origins. For instance, SARS, MERS, and COVID-19 are all caused by coronaviruses. Similarly, the H1N1 is classified as a type of Influenza A virus [4]. Zika, a viral disease transmitted through mosquito bites, is another notable example [5]. At the same time, bacteria also play a crucial role in the field of emerging infectious diseases. The emergence of mcr-1 has led to the spread of drug resistance in Gram-negative bacteria, greatly increasing the difficulty of treating bacterial infections [6]. In recent years, the emergence of new pathogenic bacteria such as Candida auris and Elizabethkingia has attracted attention [2]. These bacteria, along with viral pathogens, have created a complex landscape of emerging infectious diseases due to their unique pathogenicity and resistance mechanisms.

In tertiary general hospitals in China, nursing staff play a critical role in responding to infectious disease outbreaks. They are often the first healthcare professionals to encounter patients and are heavily involved in infection risk assessment, transmission control, and reducing infection rates [7]. However, during EID outbreaks, nursing staff frequently face immense pressure due to a lack of systematic nursing guidelines, adequate supplies, and sufficient manpower [8,9]. For example, in Taiwan, the 156th case of COVID-19 was a nurse who became infected due to insufficient infection prevention measures [10]. Given the unavoidable prolonged physical contact with patients, the competencies of nursing staff are crucial for the successful prevention and management of EIDs [11].

Competency-based training is vital for healthcare workers to effectively manage outbreaks and provide high-quality care [12]. Assessing training needs and establishing a competency index system are key steps in ensuring that healthcare workers are well prepared and capable of responding to infectious diseases [13]. The competency index system is a structured framework designed to evaluate and measure the competencies of individuals or groups within a specific work context. It consists of a series of interconnected indexes organized into three levels, progressing from broad categories to detailed evaluation criteria, ensuring precise assessment of competencies [14]. Although nurses in tertiary general hospitals play a critical role in the prevention and control of EIDs, the frameworks for assessing their ability to respond to these diseases are still inadequate. Therefore, this study aims to develop a competency index system for nurses in tertiary general hospitals to enhance their ability to respond to EIDs.

## 2. Materials and Methods

### 2.1. Design

The Delphi method was utilized to develop a competency index system for nursing staff in tertiary general hospitals in order to effectively address the challenges posed by emerging infectious diseases. This group facilitation technique, characterized by an iterative, multistage process, aims to consolidate individual opinions into a collective consensus [15]. Experts from various regions and professions were selected to participate in two rounds of electronic consultations, and we utilized a pre-prepared structured questionnaire to gather their insights on the research topic [16]. Finally, we synthesized the opinions of experts to evaluate the competency index system for nurses in tertiary general hospitals. This study was conducted according to the Guidelines for Conducting and Reporting Delphi Studies (CREDES), including the selection of a panel of experts and the design and implementation of iterative consultation rounds [17].

### 2.2. Formation of a Research Team

The research team consists of seven members, including four graduate students majoring in infectious disease nursing, one associate research fellow specializing in preventive medicine, one medical service training teacher, and one chief nurse. The tasks were divided among team members, with four graduate students and one associate research fellow handling the development and adjustment of the competency index system, while one medical service training teacher and one chief nurse oversaw the review of the competency index system and expert consultation questionnaire. Two graduate students were in charge of creating and distributing the expert consultation questionnaire. The selection of experts for the questionnaire was based on the professional experiences of the associate research fellow and chief nurse, focusing on disease prevention and control experts and medical rescue specialists with experience in COVID-19 prevention and control, such as those who worked at Wuhan Huoshenshan Hospital in China. Sorting expert opinions and analyzing data was the responsibility of four graduate students and one associate research fellow, who were split into two groups for these tasks. The first group, consisting of two graduate students, handled opinion sorting and data verification, while the second group, made up of two graduate students and one associate research fellow, was responsible for data analysis.

### 2.3. Construction of a Draft of the Competencies Index

The draft of the index system was developed through a comprehensive process involving a review of relevant literature, semi-structured interviews, and group discussions.

The literature review encompassed qualitative studies focusing on the experiences of clinical nurses and nursing managers in dealing with emerging infectious diseases, sourced from databases such as CNKI, Wanfang, CBM, PubMed, CINAHL, and Web of Science. Additionally, studies related to index systems or scales pertaining to nursing competency in the context of emerging infectious diseases were also examined. Subsequently, the research team identified the competency requirements of nursing staff and compiled a list of items and indexes related to nursing competency in the field of emerging infectious diseases.

Semi-structured interviews were conducted with eleven participants, comprising six clinical nursing managers and five clinical nurses, to further enrich the content of the index system. The interviews sought insights on the necessary knowledge, skills, attitudes, and competencies required by clinical nurses and nursing managers when engaging in rescue operations during emerging infectious disease outbreaks. Participants were also asked about the competencies needed post-outbreak and how they should prepare for future occurrences of emerging infectious diseases.

Furthermore, the research team engaged in a group discussion that incorporated various theoretical frameworks, including crisis life cycle theory, crisis management theory, and competency theory. The team examined the goals of nursing practice within the framework of responding to emerging infectious diseases. By synthesizing insights from the literature review and semi-structured interviews, the team developed a competency index system tailored for nursing personnel in tertiary general hospitals.

In the constructed system, the indexes are divided into hierarchical levels. First-level indexes are broad, covering key dimensions and integrating various competencies to guide overall evaluation. Second-level indexes refine the first-level ones with specific criteria for competency sub-components. Third-level indexes, the most detailed, provide actionable standards for accurate measurement of professional competencies [18]. This process resulted in the creation of 3 first-level indexes, 21 second-level indexes, and 118 third-level indexes.

### 2.4. Delphi Process

#### 2.4.1. Inclusion Criteria

The inclusion criteria for the consultation experts were as follows: ① worked in a tertiary general hospital or higher, and the technical title is deputy high or above; ② acquired a bachelor’s degree or above; ③ worked in the field of prevention and control of infectious diseases for >10 years, had experience in the prevention and treatment of emerging infectious diseases or public health emergencies; ④ voluntarily participated in the investigation and promised to participate in two rounds of consultation.

#### 2.4.2. First Draft of the Expert Consultation Questionnaire

The questionnaire was composed of three parts: ① a letter describing the purpose and significance of the study; ② the expert consultation form, which included 3 first-level, 21 second-level, and 118 third-level indexes. The importance of the index was evaluated using a 5-point Likert scoring method (5 = very important, 4 = important, 3 = general, 2 = unimportant, 1 =completely unimportant), and the suggestion column was provided [19]; ③ general information about experts, expert familiarity with the content of the questionnaire, and factors affecting judgment (theoretical analysis, work experience, scientific reference, and intuitive judgment) [20].

#### 2.4.3. Delphi Consulting and Feedback Cycle

**Figure 1:**
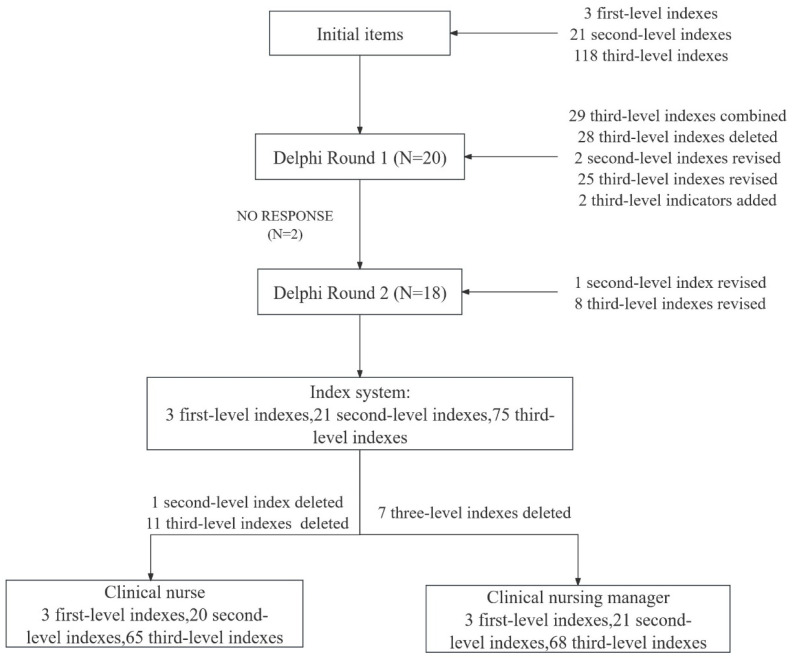
The consultation process flow chart.

From September to December 2022, the questionnaire was sent to experts by email, and two rounds of expert consultation were conducted (Figure 1). In the two rounds of correspondence, items were deleted and modified according to expert opinions and the mean importance score, the full score rate, and the coefficient of variation of each item [21].

### 2.5. Statistical Analysis

Excel 2019, SPSS 24.0, and Yaahp 10.2 software were used for data entry and analysis. The general information of the experts was expressed by frequency and composition ratio. The degree of enthusiasm of the experts was expressed as the response rate to the questionnaire. The expert authority coefficient was expressed as the coefficient of authority (CR) [22]. The degree of coordination among the experts was expressed using Kendall’s W coefficients. The evaluation of items was expressed by the mean importance score, coefficient of variation (CV), and full score rate. Index weights were calculated using the analytic hierarchy process and the average distribution method [23]. Differences were considered statistically significant at *p* < 0.05.

## 3. Results

### 3.1. General Information of Experts

A total of 18 experts from eight Chinese provinces and cities participated in the consultation for this study, including Beijing, Shanghai, Chongqing, Changsha, Guangzhou, Xi’an, Taiyuan, and Sanya. Because certain experts were active in more than one research direction or work content, the total composition of this project’s research direction was greater than 100%. General information about the experts is presented in Table 1.

### 3.2. Experts’ Enthusiasm

The enthusiasm of the experts was evaluated based on their responses to the questionnaire. In the first round of consultation, we invited 20 experts to participate, and 18 experts completed the questionnaire, with a response rate of 90%. In the second round of consultation, the response rate was 100%.

### 3.3. Expert Authority Coefficient and Opinion Coordination Degree

In the first round, the judgment, familiarity, and authority coefficients were 0.91, 0.89, and 0.90, respectively. In the second round, the judgment, familiarity, and authority coefficients were 0.90, 0.89, and 0.89, respectively. The reliability of this index system is considered high when the authority coefficient is >0.7 [24]. In the second round of expert consultation, Kendall’s W of the first-, second-, and third-level indexes were 0.564, 0.226, and 0.196 (clinical nurse), 0.239 (clinical nurse manager), respectively (Table 2).

### 3.4. Summary of Expert Consultation

The indexes were chosen based on the coefficient of variation, mean importance score, and full score. A criterion for exclusion was established, requiring that two or more conditions be simultaneously met: ① the coefficient of variation is greater than its threshold value; ② the mean importance score is less than its threshold value; ③ the full score rate is less than the threshold value [25]. The threshold values for the three evaluation indexes used to screen the competency items for nursing staff in two categories are detailed in Table 3.

In the first round of expert consultation, the research team combined 29 third-level indexes, deleted 28 third-level indexes, and modified 2 second-level indexes and 25 third-level indexes based on expert opinions and exclusion criteria. Two third-level indexes were added, namely, “Master the nursing ethical requirements in the process of prevention, control, and rescue of emerging infectious diseases” and “Be able to ensure the supply of nursing resources and the promotion of rescue behaviors in the process of multidisciplinary team cooperation”.

Based on the second round of feedback, the research team revised one second-level index and eight third-level indexes overall. Furthermore, a second-level index and eleven third-level indexes were deleted in accordance with the exclusion criteria for the clinical nurses, and seven third-level indexes were deleted in accordance with the exclusion criteria for the clinical nursing managers. The competency index system for nursing staff in tertiary general hospitals to respond to emerging infectious diseases consists of 3 first-level indexes, 21 second-level indexes, and 75 third-level indexes; of these, 20 second-level indexes and 65 third-level indexes apply to clinical nurses, while 21 second-level indexes and 68 third-level indexes apply to clinical nursing managers (Table 4 and Table 5).

### 3.5. Weight Analysis

The weights of the first- and second-level indexes were calculated using the analytic hierarchy process (AHP). The CR value of the first-level indexes was 0.051, and the CR values of the second-level indexes were 0.027, 0.023, and 0.023, respectively. When CR < 0.1, the judgment matrix was satisfactory [26]. The average distribution method (ratio of mean to total score) was used to calculate the weights of the third-level indexes [27] (Table 4 and Table 5).

## 4. Discussion

### 4.1. Scientific and Reliable

Expert selection is the key to the Delphi process. The experts selected for this study had extensive work experience in the prevention and control of infectious diseases and the management of public health emergencies. The majority of the experts, with a proportion of 77.8%, had a master’s degree or higher and came from diverse fields, such as clinical medicine, clinical nursing, nursing management, and public health. The response rates for the two rounds of consultation questionnaires in this study were 90.0% and 100%. This indicated that the experts supported this study and had great enthusiasm for participation [15]. The expert authority coefficient in the two rounds of consultations was >0.8. This indicates that the experts were familiar with the index content and could scientifically and objectively judge the entries. Kendall’s W coefficients of the first-, second-, and third-level indexes of clinical nurses and clinical nursing managers in the second round of consultation were both statistically significant. This indicates that the results of the competency index system for nursing staff in tertiary general hospitals to respond to emerging infectious diseases were reliable [28].

### 4.2. Practical Applicability

In this study, the competency index system was divided into three levels. The first-level indexes include prevention preparedness, rescue response, recovery, and reconstruction competency. Unlike other approaches that categorize knowledge, skills, and attitudes as first-level indexes, this study adopts the three-stage framework of crisis theory [29]. By aligning with the distinct stages of emerging infectious diseases, it highlights competency requirements corresponding to the full cycle of occurrence, development, epidemic spread, and disappearance of such crises [30].

The second-level index detailed the competency requirements of the nursing staff at different stages of a crisis. In the pre-crisis stage of emerging infectious diseases, the nursing staff in tertiary general hospitals need to accurately identify, scientifically assess, and effectively prevent the risks of such diseases. This aligns with Wisniewski et al.’s early risk management concept and emphasizes the importance of quickly identifying and preventing potential threats [31]. During the middle stage of the crisis, the nursing staff need to quickly identify, isolate, and respond to emerging infectious diseases [32]. The competency of epidemiological information collection and tracking, infection prevention and control, acquisition and exchange of rescue information, and multidisciplinary team cooperation are the keys to achieving these goals. This is complementary to the research on emergency care competencies of acute care medical personnel by Schultz et al. [33]. In the post-crisis stage, the nursing staff should possess the competencies of promoting physical and mental health recovery, optimizing resource allocation and facilitating the development of the nursing profession to ensure the stability of human resources and the efficient operation of the system. This requirement is highly consistent with the concept of post-disaster system recovery and resilience enhancement proposed by Polivka et al. [34], underlining the central role of nursing in post-crisis recovery.

Throughout all stages, specifically, nursing staff can utilize their risk identification and assessment capabilities to provide crucial information for clinicians’ diagnosis [35]. They can apply their information collection and tracking abilities to assist disease control personnel in grasping the epidemic transmission situation [36]. By virtue of their infection prevention and control capabilities, they can create a safe medical environment for medical staff and patients seeking treatment [37]. Relying on their multidisciplinary collaboration capabilities, they can cooperate with dietitians, respiratory therapists, etc. to ensure patients’ treatment and nutritional supply [38]. Furthermore, for nursing administrators, the detailed content of the competency indexes is conducive to the application of the index system in the training and evaluation of nursing staff competencies [39]. Based on the basic principles of competency theory, third-level indexes were developed to describe the connotations and constituent aspects of second-level competency indexes [40].

### 4.3. Difference and Specificity

The study delineated the competency indexes for clinical nurses and clinical nursing managers to respond to emerging infectious diseases, as informed by expert consultations. Unlike clinical nurses, the competency indexes for clinical nursing managers primarily encompass policy formulation, system development, program recommendations, and organizational resource coordination and management: for instance, the ability to propose nursing response policies for emerging infectious diseases, establish commendation policies to support staff well-being programs, ensure the provision of nursing resources, and promote collaborative rescue efforts within multidisciplinary teams. Previous research by Gebbie KM and Qureshi K expanded on managerial and leadership competencies, including planning and gap-filling skills, such as ensuring the existence and regular practice of emergency response plans [41]. In the executive research on nursing leaders by Tarja Jääski et al., it is also indicated that nurse leaders require new and diverse crisis management competencies. These competencies include but are not limited to interactive communication competency, psychological resource management competency, and systematic and proactive organizing competency [42].

Moreover, the significance of the same competencies varied between the two groups. The distinctiveness and specificity of competencies required by clinical nurses and clinical nursing managers to respond to emerging infectious diseases primarily stem from their respective job roles [7]. Clinical nursing managers engage with a wider array of stakeholders compared to clinical nurses, including clinical nurses, patients, other medical supervisors collaborating with the nursing team, and senior management [43]. Additionally, the nature of their work differs, with clinical nurses primarily focused on patient care, while clinical nursing managers are tasked with overseeing the coordinated delivery of nursing services within departments or wards and ensuring overall service quality control [44].

Considering the differences in competencies required by clinical nurses and nursing managers, as well as the unpredictability of emerging infectious diseases, comprehensive training is essential. In addition to basic theoretical and skills training, emergency drills are an effective way to assess and maintain skills. For instance, some hospitals in China conduct full-hospital emergency drills 2–3 times a year, tailored to various emergency events [45]. These drills are crucial, ensuring that nursing staff and healthcare workers are prepared to respond quickly and effectively in crises [46].

### 4.4. Limitations

This study had some limitations. Firstly, the experts were from eight provinces or municipalities in China, which may not be representative. Thus, the results may have some regional bias, which may limit their generalizability. Secondly, Kendall’s W values vary across different levels of indexes. The first-level index shows high consistency, while the second and third levels have lower consistency. This might be due to the complexity or diversity of specific skills and roles, causing experts to have differing views at detailed levels. Subsequent studies would further explore the reasons and optimization methods. Thirdly, an empirical study of the index system has not yet been conducted, and its validity and applicability require further testing.

## 5. Conclusions

Through two rounds of expert consultation, the competency index system for nurse staff to respond to emerging infectious diseases was established, which is reliable and scientific and can provide a reference for training nursing competency and selecting nursing talents to respond to emerging infectious diseases. Further empirical research on this index system will be conducted in the future.

## Figures and Tables

**Table 1:** General information about the experts.

Variable	Frequency	Proportion (%)
Age (years)		
40~	9	50.0
50~	9	50.0
Sex		
Women	16	88.9
Men	2	11.1
Educational background		
Bachelor	4	22.2
Master	7	38.9
Doctor	7	38.9
Title		
Vice-senior	3	16.7
Senior	15	83.3
Working experience (years)		
20~	1	5.6
30~	15	83.3
40~	2	11.1
Research field		
Healthcare management	13	72.2
Infectious disease nursing	6	33.3
Emergency and critical nursing	6	33.3
Disease prevention and control	6	33.3

**Table 2:** The result of the degree of coordination of experts’ opinions.

Round	Level		Kendall’s W	χ ^2^	*p*
First	First-level index		0.492	25.079	0.093
	Second-level index		0.298	106.458	<0.001
	Third-level index	Clinical nurse	0.069	139.052	<0.001
		Clinical nursing manager	0.394	790.647	<0.001
Second	First-level index		0.564	28.769	0.037
	Second-level index	Clinical nurse	0.234	75.447	<0.001
		Clinical nursing manager	0.226	76.846	<0.001
	Third-level index	Clinical nurse	0.196	213.289	<0.001
		Clinical nursing manager	0.239	275.880	<0.001

Kendall’s W, Kendall’s coefficient of concordance. χ^2^, chi-square statistic.

**Table 3:** The threshold value of the mean, the coefficient of variation, and the full score.

Type	Variable	Threshold Value	Average	SD
Clinical nurse	Mean	3.862	4.485	0.623
	Full score	0.772	0.897	0.125
	CV	0.296	0.143	0.153
Clinical nursing manager	Mean	4.750	4.877	0.127
	Full score	0.950	0.975	0.025
	CV	0.131	0.066	0.065

CV, Coefficient of variation. SD, Standard deviation.

**Table 4:** Consultation results for the first-level and second-level indexes of the competency index system for nursing staff in tertiary general hospitals to respond to EIDs (the second round).

Item	Significance Grade $(\bar{X}\pm S$)	CV	Clinical Nurse	Clinical Nursing Manager
			Weight	Weight
I Prevention and Preparedness	4.94 ± 0.24	0.048	0.311	0.311
I-1 Clear roles and responsibilities	4.83 ± 0.38	0.079	0.133	0.106
I-2 Policy implementation and improvement	4.72 ± 0.46	0.098	0.091	0.075
I-3 Protection and treatment resources prepared	4.89 ± 0.32	0.066	—— *	0.150
I-4 Infectious diseases knowledge and skills updating	4.89 ± 0.32	0.066	0.174	0.150
I-5 Surveillance and early warning	4.89 ± 0.32	0.066	0.174	0.150
I-6 Infection risk assessment and prevention	4.94 ± 0.24	0.048	0.250	0.222
I-7 Infectious disease information management	4.78 ± 0.55	0.115	0.110	0.089
I-8 Mental adjustment	4.67 ± 0.59	0.127	0.068	0.060
II Respond and Rescue	5.00 ± 0.00	0.000	0.493	0.493
II-1 Infection prevention and control	5.00 ± 0.00	0.000	0.172	0.172
II-2 Rescue area management	4.94 ± 0.24	0.048	0.121	0.121
II-3 Rescue information acquisition and exchange	4.89 ± 0.32	0.066	0.104	0.104
II-4 Rescue resource replenishment and dispatch	4.83 ± 0.38	0.079	0.082	0.082
II-5 Epidemiologic information collection and tracking	4.50 ± 0.51	0.114	0.039	0.039
II-6 Psychological crisis prevention and treatment	4.72 ± 0.58	0.122	0.055	0.055
II-7 Multidisciplinary team collaboration	4.83 ± 0.38	0.079	0.082	0.082
II-8 Nursing intervention	5.00 ± 0.00	0.000	0.172	0.172
II-9 Emergency care	5.00 ± 0.00	0.000	0.172	0.172
III Recovery and Reconstruction	4.83 ± 0.51	0.106	0.196	0.196
III-1 Physical and mental health rehabilitation	4.89 ± 0.32	0.066	0.393	0.393
III-2 Nursing resource optimization	4.78 ± 0.43	0.090	0.234	0.234
III-3 Nursing professional development	4.78 ± 0.55	0.115	0.234	0.234
III-4 Dynamic tracking of the development of EIDs	4.72 ± 0.46	0.098	0.139	0.139

$\bar{X}$, mean value. S, standard deviation. CV, Coefficient of variation. * There are no third-level indexes for the clinical nurses in item I-3, and they were not granted weight values.

**Table 5:** Consultation results for the third-level indexes of the competency index system for nursing staff in tertiary general hospitals to respond to EIDs (the second round).

Item	Clinical Nurse			Clinical Nursing Manager		
	Significance $\mathrm{Grade}\;(\bar{X}\pm S$)	CV	Weight	Significance $\mathrm{Grade}\;(\bar{X}\pm S$)	CV	Weight
I-1-1 Comply with national/hospital/ward the responsibility of nursing staff for EIDs	4.89 ± 0.32	0.066	0.344	4.89 ± 0.32	0.066	0.497
I-1-2 Describe the roles and responsibilities of hospitals, wards, and nurses for EIDs	4.67 ± 0.77	0.164	0.328	4.94 ± 0.24	0.048	0.503
I-1-3 Be able to carry out health education on the prevention and control of infectious diseases	4.67 ± 0.49	0.104	0.328	——		
I-2-1 Understand the laws and regulations related to the prevention, control, and treatment of infectious diseases, public health emergencies, and biosafety incidents	4.78 ± 0.55	0.115	0.326	5.00 ± 0.00	0.000	0.252
I-2-2 Be familiar with the emergency plan for EIDs in the hospital/ward	5.00 ± 0.00	0.000	0.341	5.00 ± 0.00	0.000	0.252
I-2-3 Master the nursing ethical requirements in the process of prevention, control, and rescue of EIDs	4.89 ± 0.32	0.066	0.333	4.94 ± 0.24	0.048	0.249
I-2-4 Be able to recommend policy options for nursing response to EIDs.	——			4.89 ± 0.32	0.066	0.246
I-3-1 Set up emergency nursing echelon team for EIDs	——			4.72 ± 0.46	0.098	0.488
I-3-2 Be able to allocate nursing facilities and equipment and stock supplies according to the needs of medical tasks and nursing responsibilities	——			4.94 ± 0.24	0.048	0.512
I-4-1 Be qualified and able to complete the training and drills program of infectious disease prevention and control	4.94 ± 0.24	0.048	1.000	5.00 ± 0.00	0.000	1.000
I-5-1 Be familiar with the definition of infectious disease symptom surveillance or single disease surveillance	4.83 ± 0.38	0.079	0.335	4.94 ± 0.24	0.048	0.500
I-5-2 Be familiar with the syndromes, transmission routes, and epidemiological characteristics of infectious diseases	4.94 ± 0.24	0.048	0.342	4.94 ± 0.24	0.048	0.500
I-5-3 Be familiar with the reporting system and procedure for infectious diseases in the hospital	4.67 ± 1.19	0.255	0.323	——		
I-6-1 Be familiar with the standard precautionary principles and protective equipment	5.00 ± 0.00	0.000	0.252	5.00 ± 0.00	0.000	0.250
I-6-2 Be able to assess and avoid the risk of infection in the nursing environment and nursing behaviors	4.94 ± 0.24	0.048	0.249	5.00 ± 0.00	0.000	0.250
I-6-3 Master the measures of isolation, decontamination, quarantine, and sewage (including environmental disinfection, specimen collection, donning and doffing of personal protective equipment, the disposal of medical waste, etc.).	4.89 ± 0.32	0.066	0.246	5.00 ± 0.00	0.000	0.250
I-6-4 Master the emergency treatment of occupational exposure of the respiratory tract, blood, body fluids, etc.	5.00 ± 0.00	0.000	0.252	5.00 ± 0.00	0.000	0.250
I-7-1 Have awareness of information security protection	4.78 ± 0.43	0.090	0.371	4.89 ± 0.32	0.066	0.333
I-7-2 Be able to record and file information on the procedures, materials, and personnel arrangements of the nursing staff in the ward to respond to infectious diseases or emergencies	4.22 ± 1.22	0.288	0.328	4.83 ± 0.38	0.079	0.330
I-7-3 Be able to analyze and interpret infectious disease and emergency data and assess the nursing response to risk	3.89 ± 1.57	0.403	0.302	4.94 ± 0.24	0.048	0.337
I-8-1 Be familiar with the personal psychological defense line for stress and stress relief	4.78 ± 0.43	0.090	0.341	4.94 ± 0.24	0.048	0.338
I-8-2 Be able to help hospital/ward personnel understand infectious diseases correctly and overcome fear	4.56 ± 0.62	0.135	0.325	4.83 ± 0.38	0.079	0.331
I-8-3 Master the psychological nursing techniques and relieve the adverse emotional reaction of patients/colleagues by relieving, guiding, or encouraging	4.67 ± 0.49	0.104	0.333	4.83 ± 0.38	0.079	0.331
II-1-1 Master the basic knowledge of the EID, such as symptoms, signs, transmission routes, and epidemiological characteristics	5.00 ± 0.00	0.000	0.200	5.00 ± 0.00	0.000	0.200
II-1-2 Master the personal protective equipment application requirements for the EID	5.00 ± 0.00	0.000	0.200	5.00 ± 0.00	0.000	0.200
II-1-3 Master the methods of source isolation of the EID	5.00 ± 0.00	0.000	0.200	5.00 ± 0.00	0.000	0.200
II-1-4 Master the standard of the EID decontamination	4.94 ± 0.24	0.048	0.198	5.00 ± 0.00	0.000	0.200
II-1-5 Master the emergency treatment methods and procedures for the failure of protection and suspected or occupational exposure	5.00 ± 0.00	0.000	0.200	5.00 ± 0.00	0.000	0.200
II-2-1 Be able to lay out space for the EID in the ward in accordance with the latest prevention and control guidelines	4.50 ± 1.25	0.278	0.238	5.00 ± 0.00	0.000	0.251
II-2-2 Master the ward admission and visiting service system for the EID	5.00 ± 0.00	0.000	0.265	5.00 ± 0.00	0.000	0.251
II-2-3 Be familiar with the plan for the management of the medicine and protective materials in the responsible area and maintain the normal operation of the equipment	4.56 ± 0.62	0.135	0.241	5.00 ± 0.00	0.000	0.251
II-2-4 Master the ways of collecting and sending samples of environmental surface and air microorganism detection	4.83 ± 0.51	0.106	0.256	4.89 ± 0.32	0.066	0.246
II-3-1 Be able to identify the correct way to obtain up-to-date ambulance information and be alert to the transmission and dissemination of unconfirmed ambulance news	4.72 ± 0.46	0.098	0.263	4.89 ± 0.32	0.066	0.200
II-3-2 Be able to communicate with hospital/ward staff smoothly and quickly	4.39 ± 1.20	0.272	0.245	4.94 ± 0.24	0.048	0.202
II-3-3 Be able to ensure the transparency of nursing staff to grasp the latest information of the EID	——			4.94 ± 0.24	0.048	0.202
II-3-4 Be able to carry out health education to the patients and provide information on diagnosis, treatment, and protection of the EID	4.67 ± 0.49	0.104	0.260	4.89 ± 0.32	0.066	0.200
II-3-5 Understand the ways to seek rescue help and support from the community	4.17 ± 1.25	0.300	0.232	4.83 ± 0.38	0.079	0.197
II-4-1 Be able to apply all kinds of prevention and control materials, treatment materials, and living materials according to the standard of job requirement	4.67 ± 0.49	0.104	1.000	4.94 ± 0.24	0.048	0.331
II-4-2 Be familiar with the material consumption and reserves and be able to communicate with the logistics department in time to supplement the materials	——			5.00 ± 0.00	0.000	0.335
II-4-3 Master nursing manpower on-the-job situation, coordinate the corresponding nursing manpower	——			5.00 ± 0.00	0.000	0.335
II-5-1 Be familiar with the scope of epidemiological survey data for the EID	4.06 ± 0.87	0.215	0.303	——		
II-5-2 Master the knowledge and skills of sample collection and transportation in an epidemiological survey	4.50 ± 0.86	0.190	0.336	——		
II-5-3 Be able to know the significance of epidemiological survey results and to correctly shunt patients	4.83 ± 0.38	0.079	0.361	4.83 ± 0.38	0.079	1.000
II-6-1 Have a stable psychological state to respond to the EID	4.89 ± 0.32	0.066	0.257	4.89 ± 0.32	0.066	0.251
II-6-2 Have the consciousness of propagating positive nursing deeds to enhance the sense of achievement	4.61 ± 0.50	0.109	0.243	4.83 ± 0.38	0.079	0.248
II-6-3 Be familiar with the state of psychological stress and detect the first signs of possible psychological crisis in patients/colleagues/self	4.72 ± 0.46	0.098	0.249	4.89 ± 0.32	0.066	0.251
II-6-4 Master nursing intervention methods for psychological crisis and seek help from a professional psychological assistance team when necessary	4.78 ± 0.43	0.090	0.251	4.89 ± 0.32	0.066	0.251
II-7-1 Be able to work as a team	4.89 ± 0.32	0.066	0.339	5.00 ± 0.00	0.000	0.253
II-7-2 Be familiar with the roles and tasks of others in the multidisciplinary team	4.56 ± 0.62	0.135	0.331	4.94 ± 0.24	0.048	0.250
II-7-3 Be able to co-negotiate rescue plan to ensure smooth communication and effective cooperation among multidisciplinary teams	4.44 ± 0.62	0.139	0.331	4.94 ± 0.24	0.048	0.250
II-7-4 Be able to ensure the supply of nursing resources and the promotion of rescue behaviors in the process of multidisciplinary team cooperation	——			4.89 ± 0.32	0.066	0.247
II-8-1 Have predictive nursing thinking	4.83 ± 0.38	0.079	0.199	4.94 ± 0.24	0.048	0.201
II-8-2 Be able to take a complete history and assess the health status and nursing needs of patients with the EID	5.00 ± 0.00	0.000	0.206	4.89 ± 0.32	0.066	0.199
II-8-3 Be able to clarify nursing diagnoses based on critical thinking in conjunction with a multidisciplinary team plan of care	4.78 ± 0.43	0.090	0.197	4.89 ± 0.32	0.066	0.199
II-8-4 Be able to develop and implement evidence-based individualized nursing plans	4.94 ± 0.24	0.065	0.204	4.89 ± 0.32	0.066	0.199
II-8-5 Be able to join with members of the multidisciplinary team to judge the effectiveness of nursing response behaviors and make timely adjustments to the plan of nursing	4.72 ± 0.58	0.050	0.195	4.94 ± 0.24	0.048	0.201
II-9-1 Be familiar with the clinical manifestations of mild, moderate, and severe patients with the EID per the latest guidelines	4.94 ± 0.24	0.048	0.167	4.94 ± 0.24	0.048	0.166
II-9-2 Master the dose, concentration, method, and time of medicine for the treatment of the EID per the latest guidelines	4.94 ± 0.24	0.048	0.167	4.94 ± 0.24	0.048	0.166
II-9-3 Be able to apply emergency medical equipment while wearing protective gear, as required by the job.	5.00 ± 0.00	0.000	0.169	5.00 ± 0.00	0.000	0.168
II-9-4 Be able to perform general nursing measures, such as life nursing, skin nursing, pipeline nursing, etc., for patients while wearing protective equipment	4.72 ± 1.18	0.252	0.159	4.94 ± 0.24	0.048	0.166
II-9-5 Be able to administer basic first aid to the patient under the requirements of level II protection, such as oxygen, sputum, arterial puncture, cardiopulmonary resuscitation (CPR), gastric lavage, and urinary catheterization	5.00 ± 0.00	0.000	0.169	4.94 ± 0.24	0.048	0.166
II-9 -6 Master the method and process of hospital transportation for patients with the EID	5.00 ± 0.00	0.000	0.169	5.00 ± 0.00	0.000	0.168
III-1-1 Be able to provide continuity of care to meet the physical and psychological care needs of infected patients after discharge from the hospital	4.39 ± 1.20	0.139	0.479	——		
III-1-2 Be able to recuperate or cooperate with physical and mental recovery treatment programs until return to daily nursing duties	4.78 ± 0.43	0.080	0.521	4.83 ± 0.38	0.079	0.503
III-1-3 Be able to develop a nursing commendation policy to support a physical and mental adjustment program for nursing staff	——			4.78 ± 0.43	0.090	0.497
III-2-1 Be able to improve and optimize personal resources such as infectious disease knowledge reserve, emergency practice skill proficiency, and professional identity	4.00 ± 1.61	0.096	0.335	4.83 ± 0.38	0.079	0.250
III-2-2 Be able to improve the nursing supplies and human resources reserve plan for EIDs in the ward	——			4.89 ± 0.32	0.066	0.253
III-2-3 Be able to supplement the content of hospital/ward nursing education and training resources for EIDs	3.94 ± 1.26	0.117	0.330	4.78 ± 0.43	0.090	0.247
III-2-4 Be able to review and analyze lessons learned in responding to EIDs and optimize nursing plans, systems, and policies	4.00 ± 0.84	0.096	0.335	4.83 ± 0.38	0.079	0.250
III-3-1 Be able to share and assimilate the excellent nursing experience of EIDs	4.61 ± 0.61	0.083	0.525	4.83 ± 0.38	0.079	0.503
III-3-2 Be able to conduct nursing field studies for EIDs-affected population	4.17 ± 0.71	0.146	0.475	——		
III-3-3 Be able to develop a training program on nursing competence for EIDs	——			4.78 ± 0.43	0.090	0.497
III-4-1 Be able to continuously implement the monitoring, early warning, and reporting of existing cases of EIDs	4.22 ± 1.26	0.136	0.475	——		
III-4-2 Be able to update and learn new guidelines for the prevention and control of EIDs	4.67 ± 0.59	0.092	0.525	4.78 ± 0.43	0.090	0.497
III-4-3 Be able to dynamically adjust the nursing plan for EIDs in the ward in conjunction with the national infectious disease prevention policy	——			4.83 ± 0.38	0.079	0.503

$\bar{X}$, mean value. S, standard deviation. CV, Coefficient of Variation.

## Data Availability

Data are contained within the article.
